# He Rourou Whai Painga, an Aotearoa New Zealand dietary pattern for metabolic health and whānau wellbeing: protocol for a randomized controlled trial

**DOI:** 10.3389/fnut.2023.1298743

**Published:** 2023-12-11

**Authors:** Fiona E. Lithander, Amber Parry Strong, Andrea Braakhuis, Anna Worthington, Meika Foster, Anna Rolleston, Cheryl Davies, Jane Mullaney, Cecilia Ross, Denise Conroy, Troy L. Merry, Richard Gearry, Mark Weatherall, Jeremy D. Krebs

**Affiliations:** ^1^New Zealand National Science Challenge High Value Nutrition, Liggins Institute, University of Auckland, Auckland, New Zealand; ^2^Discipline of Nutrition, School of Medical Sciences, The University of Auckland, Auckland, New Zealand; ^3^The Liggins Institute, The University of Auckland, Auckland, New Zealand; ^4^Centre for Endocrine, Diabetes and Obesity Research, Te Whatu Ora New Zealand Capital, Coast and Hutt Valley, Wellington, New Zealand; ^5^Edible Research Ltd., Christchurch, New Zealand; ^6^Centre for Health, Manawa Ora Centre, Tauranga, New Zealand; ^7^Tū Kotahi Māori Asthma and Research Trust, Kōkiri Marae, Lower Hutt, New Zealand; ^8^The New Zealand Institute for Plant & Food Research Ltd., Auckland, New Zealand; ^9^Maurice Wilkins Centre for Molecular Biodiscovery, The University of Auckland, Auckland, New Zealand; ^10^Department of Medicine, University of Otago, Christchurch, New Zealand; ^11^Department of Medicine, University of Otago, Wellington, New Zealand

**Keywords:** metabolic syndrome, dietary pattern, mediterranean diet, cardiometabolic, metabolic syndrome severity score, behavior change support, household/family intervention

## Abstract

**Background:**

Cardiometabolic diseases are highly prevalent in Aotearoa New Zealand. Dietary intake is a modifiable risk factor for such diseases and certain dietary patterns, specifically the Mediterranean diet (MedDiet), are associated with improved metabolic health. This study aims to test whether an intervention including a Mediterranean dietary pattern incorporating high quality New Zealand foods (NZMedDiet pattern) and behavior change science can improve the metabolic health of participants and their household/whānau.

**Methods and analysis:**

This is a multi-center, three-stage trial with two parallel group superiority randomized controlled trials (RCTs), and a longitudinal cohort study embedded within the trial design. The first RCT (RCT 1) is a comparison of the NZMedDiet pattern compared to usual diet for 12 weeks. The Behavior Change Wheel was used to select and implement strategies to support participant adherence to the NZMedDiet, such as web-based nutrition education on healthy shopping and cooking. The second (RCT 2) compares online social support to no online social support for 12 weeks, administered to participants immediately following RCT 1. The third stage is a longitudinal cohort study where all participants are followed from the beginning of their start of the active intervention for 12 months in total. The primary outcome measure for each stage is the metabolic syndrome severity score (MetSSS). The duration of enrolment is 12–15 months. The total recruitment target is 200 index participants and their household/whānau members who participate with them, and the primary analyses will be intention to treat on index participants.

**Discussion:**

The trial will test whether the NZMedDiet pattern and behavior change support improves the cardiometabolic health of people in Aotearoa New Zealand.

**Clinical trial registration:**

https://www.anzctr.org.au/Default.aspx, identifier ACTRN12622000906752 and https://www.isrctn.com/, identifier ISRCTN89011056 (Spirit 2).

## Introduction (Spirit 6a)

Cardiometabolic diseases, including type 2 diabetes (T2DM) and cardiovascular disease, are common. In 2017, one in three deaths were caused by cardiovascular disease in Aotearoa New Zealand (1). Diet is an important modifiable risk factor for cardiovascular disease (2), and certain dietary patterns such as the Mediterranean diet (MedDiet) are associated with a reduced risk of cardiometabolic disease (3, 4). The MedDiet is rich in minimally processed plant-based foods and legumes; low in saturated fat and red meat; and a distinguishing feature is its use of olive oil (3). Nutrition knowledge regarding the benefits of adopting a dietary pattern such as the MedDiet does not always translate into a change in dietary behavior. Dietary change can be affected by barriers to behavior change such as cost, acceptability, time, dietary knowledge, nutrition competence and cultural practices (5).

Recent research from our group, and others, reports that the use of, and adherence to, a MedDiet pattern in Aotearoa New Zealand is low (6, 7). To promote a MedDiet pattern in countries such as Aotearoa New Zealand, attention should be given to optimizing a dietary intervention, taking into consideration the behavioral and social structures that decrease barriers to adherence (8, 9). Studies of interventions aimed at improving dietary patterns in community living individuals report that barriers to a healthier diet include the “good taste” of unhealthy food, inconvenience and difficulties in enjoying non-fatty food (5, 10). Individual choice, cultural norms and taste preferences of the study population are important to consider when adapting a dietary pattern into a population. Studies recruiting Māori, who are indigenous New Zealanders, and young adults of various ethnicities, report that the provision of meal preparation support and financial assistance for the use of healthy diets, may be useful to support changes in dietary patterns (11, 12). Food provision, barriers to which may have otherwise prevented dietary change, such as the lack of prior exposure, uncertainty about how to prepare the food, and cost, is a potential intervention that may enable the uptake of a healthier diet (13). The social context of sharing and consuming meals within a household/whānau is an important aspect of many cultures and takes into consideration that all the household/whānau members within a dietary intervention may facilitate ongoing dietary change for all (12, 14, 15). The provision of food and related education is reported to support adherence to a healthy diet, and lower the burden associated with food preparation, particularly when applied on a household/whānau basis (16). The behavior change wheel framework is underpinned by the Capability, Opportunity and Motivation Behavior (COM-B) theory and facilitates consideration of the aforementioned barriers to adopting the desired dietary pattern (17, 18). Consequently, this framework will be used to identify effective and feasible strategies to overcome these and support participant adherence.

The primary objective of this trial is to evaluate if a Mediterranean dietary pattern incorporating high quality New Zealand foods (NZ MedDiet pattern), delivered in a household/whānau setting and underpinned by behavior science can improve the cardiometabolic health of people in Aotearoa New Zealand. We have chosen to do this through the metabolic syndrome severity score (MetSSS). The metabolic syndrome (MetS) refers to the clustering of several risk factors including hypertension, obesity, dyslipidemia and insulin resistance, which identifies individuals at greater risk of CVD and T2DM (19). MetS is commonly classified in a dichotomous fashion, as having or not having MetS. An alternative approach, which gives a continuous and more nuanced description, is the Metabolic Syndrome Severity Score (MetSSS) which quantifies the value of MetS latent factors for an individual, and the resulting score behaves like a Z-score in that it is normally distributed in a population. The MetSSS has been shown to predict future cardiometabolic disease and can be modified by diet, exercise, and pharmacological intervention to estimate change in cardiometabolic disease risk, and is therefore a useful tool in assessing the impact of a dietary intervention (19). It has been validated in a New Zealand population and has been shown to be responsive to diet and lifestyle interventions in people with pre-diabetes (19–21).

A secondary objective of this study is to evaluate the effectiveness of continued online social support (Spirit 7).

## Methods and analysis

This protocol is reported in accordance with the SPIRIT (Standard Protocol Items: Recommendations for Interventional Trials) guidance (22). In preparation for this trial, a feasibility study was conducted to test the recruitment and screening strategy, retention, acceptability of the method of delivery of a component of the intervention, and to collect relevant data to refine the power calculation for this study (23).

### Trial design (Spirit 8)

**Figure 1:**
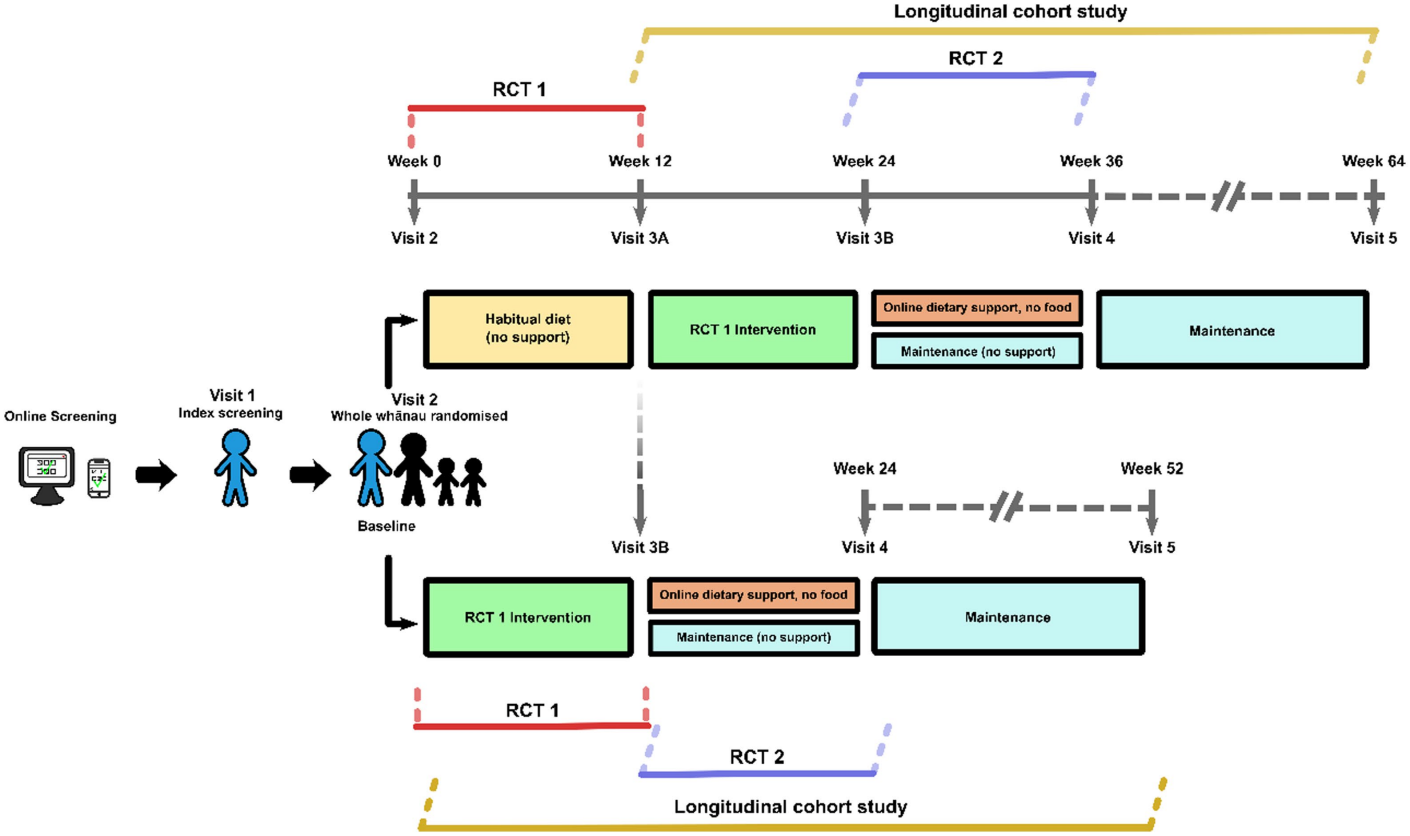
Study design.

This is a multi-center, three-stage trial, with two randomized controlled trials (RCTs); both parallel group superiority trials, and a longitudinal cohort study. The first RCT (RCT 1) compares the effect of Mediterranean dietary pattern incorporating high quality New Zealand foods (NZMedDiet pattern) to usual diet on MetSSS, a marker of cardiometabolic health for 12-weeks. The intervention in RCT 1 includes elements of behavior change science including the provision of food, recipes, weekly meal plans, access to web-based nutrition education and opt-in online social support. From here on within the manuscript ‘RCT 1 intervention’ is referred to as a package including these described aspects. The second RCT (RCT 2) compares the opt-in online social support, to no online social support with an outcome assessment 12 weeks after the second randomization (Figure 1). Those participants who were originally randomized to the control group in RCT 1 will be given the RCT 1 intervention before the second randomization. After the second randomization, both groups continue to have access to the bespoke study website housing nutrition information and education. The third stage is a longitudinal cohort study where all participants are followed for 12 months from the start of the dietary intervention. The trial design is summarized in Figure 1 (Spirit 8).

### Trial setting (Spirit 9)

The trial settings are four centers in Aotearoa New Zealand: two based at university research units (University of Auckland and University of Otago, Christchurch), one at a community-based traditional Māori meeting place (Tū Kotahi Māori Asthma and Research Trust at Kōkiri Marae in Lower Hutt, Wellington), and the other based at a hospital-based research unit (the Center for Endocrine Diabetes and Obesity Research (CEDOR) in Wellington).

### Eligibility criteria (Spirit 10)

**Figure 2:**
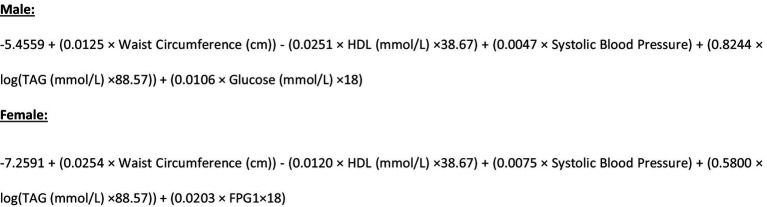
Formulae used for the calculation of the MetSSS.

Index participants and their household/whānau members will be recruited through the four trial sites. Multiple recruitment strategies will be used to recruit participants to the study. Advertising to the public through television, newspaper and radio advertisements, study posters, study information sessions, and other hardcopy and online media, including targeted social media, and institutional mailing lists will be used. Participants will be recruited from the local populations of the area of each study center. An important element of recruitment for research in Aotearoa New Zealand is to enhance Māori capacity and capability for research, and to include Māori and their whānau as research participants. This will be enabled by the close relationship between CEDOR and Kōkiri Marae to support the staff at Kōkiri Marae and oversee the running of the trial at that site (Figure 2).

Tables 1, 2 show the eligibility criteria for both index participants and household/whānau participants. Screening will occur consistent with our feasibility study (23). Potential participants who respond to advertisements or who otherwise contact researchers will be directed to the trial website^1^ and an on-line questionnaire to complete as step one of the two step screening process. The website provides information about the trial and the questionnaire includes contact information and questions to enable calculation of the AUSDRISK (24). If this score is greater than 12, potential participants will be invited to a second step of screening which is conducted in-person at one of the study sites. This will include a fasting blood sample and anthropometric measurements to enable the calculation of the MetSSS. If they have a fasted MetSSS greater than 0.35, they will be the index participant. Once identified, and having given written consent for participating, household/whānau members of the index participant will also be invited to participate in the trial. Between one and five household/whānau members per index participant will be invited. The food provision intervention (see below) will be calculated based on the total number of people in the household, for up to 6 members. All household/whānau members who qualify for inclusion will complete age-appropriate questionnaires (Table 3), will be invited to undertake clinical measurements and to provide blood samples if aged 11 years and over. If an index participant and household/whānau participant were to separate over the course of the trial, attempts would be made to gather data from those who continue living in the same building as the index participant.

**Table 1:** Inclusion and exclusion criteria for index participants.

Inclusion criteria	Exclusion criteria
18–70 years	Previous bariatric surgery, or pre-existing Type 1, or Type 2 diabetes. Where a previous diagnosis of T2DM is uncertain, this will be defined as ever having had two consecutive HbA1c results ≥50 mmol/mol that are at least three months apart
Metabolic syndrome severity Score (MetSSS) >0.35	Total cholesterol ≥8 mmol/L
1–5 household/whānau members agree to participate*	Chronic severe renal disease (eGFR <30 mL/min/1.72m^2^)
Participants and their whānau are planning to live together for the duration of the study	Current pregnancy or breastfeeding, or planning to conceive during the study (due to impact on interpreting outcome measures)
Access to the internet at home	Unstable body weight (active weight loss/gain >5 kg in prior three months)
Able and willing to attend all site visits	Gastrointestinal disorder that alters the digestion and absorption of nutrients (e.g., ulcerative colitis, Crohn’s disease, coeliac disease, an ileostomy or colostomy).
Willing to adhere to local health and safety regulations	Severe food allergies (anaphylaxis) in any household/whānau member
For the consumer insights study, there needs to be a willingness to be interviewed	Medication use – current use of medications that modify blood sugar levels, or anticipated regular use of oral or injected steroids
	Does not agree to refrain from donating blood for three months prior to each study visit (due to impact on HbA1c)
	Is participating in, or has recently participated in another research study involving an intervention which may alter outcomes of interest to this study
	Any other condition or situation, which in the view of investigators would affect the compliance or safety of the individual taking part

^*^Additional whānau/household members, if they are eligible and interested, may also be enrolled as an index participant.

**Table 2:** Inclusion criteria for household/whānau participants and exclusion criteria for household/whānau participants who provide a blood sample.

Living in the same household as the index individual	Age ≤ 11 years
Consent/assent to consume the intervention food	Pre-existing Type 1 diabetes.
	Chronic severe renal disease (eGFR <30 mL/min/1.72m^2^)
	Current pregnancy or breastfeeding, or planning to conceive during the study (due to impact on interpreting outcome measures)
	Unstable body weight (active weight loss/gain >5 kg in prior three months)
	Gastrointestinal disorder that alters the digestion and absorption of nutrients (e.g., ulcerative colitis, Crohn’s disease, coeliac disease, an ileostomy or colostomy).
	Medication use – current use of medications that modify blood sugar levels, or anticipated regular use of oral or injected steroids
	Does not agree to refrain from donating blood for three months prior to each study visit (due to impact on HbA1c)
	Is participating in, or has recently participated in another research study involving an intervention which may alter outcomes of interest to this study
	Any other condition or situation, which in the view of investigators would affect the compliance or safety of the individual taking part
	Children living in the household but who do not have a legal guardian also living in the household

**Table 3:** Summary of questionnaires.

	How administered	What it measures	Which participants
Otago short-form FFQ (25)	On REDCap	Habitual dietary intake	All index participants and household/whānau participants aged 11y and over
Three factor eating questionnaire or child version (26)	Emailed survey which will be linked to REDCap	Cognitive restraint uncontrolled eating and emotional eating	All index participants and household/whānau participants aged 11y and over
24 h Food recall	On intake 24 website (https://intake24.co.uk/info/recall)	Dietary intake in previous 24 h	All index participants and household/whānau participants aged 11y and over
IPAQ Physical activity (27)	On REDCap	Habitual physical activity	All index participants and household/whānau participants aged 11y and over
SF 36 (28)	On REDCap	Quality of life	All index participants and household/whānau participants aged 11y and over
Kaupapa Māori Wellbeing questionnaire	On REDCap	Covering the four realms of Te Whare Tapa Whā to determine quality of life from a Māori perspective	Any Māori participants aged 11y and over
Gastrointestinal symptom rating scale (29)	On REDCap	Gastrointestinal symptoms	All index participants and household/whānau participants aged 11y and over
Self-evaluation COM Questionnaire (30)	On REDCap	Evaluates the process; assesses participant perceptions of barriers and enablers throughout the study to see if the behavior change support is working as it is intended	Index participants
Impact Evaluation for Nutrition and Behavior Change Support (31)	On REDCap	Assesses how the behavior change support impacts participants (including engagement, satisfaction, and perception of impact)	Index participants

FFQ, food frequency questionnaire; IPAQ, international physical activity questionnaire; COM, capability opportunity motivation.

### Who will take informed consent (Spirit 26a)

Written informed consent will be obtained from index participants at Visit 1 by a member of the study team and from whānau/household participants at Visit 2 (Figure 1). Assent and parental/caregiver consent will be collected from household/whānau participants who are aged less than 16 years. However, if a 14- or 15-year-old child demonstrates an understanding of what the study involves at Visit 2, they will be invited to complete consent. Additional written consent will be requested from index participants at visit 1 for several sub-studies. These include (1) participation in a mixed meal tolerance test at two timepoints (2) participation in a sub-study around the measurement of dietary intake using a phone application. In addition, at visit 1, index participants will be asked if they wish to learn more about a qualitative study that runs in parallel with the current study. If so, their contact details will be passed to a qualitative researcher who will contact them by phone with further details and will take consent, as applicable.

### Additional consent provisions for collection and use of participant data and biological specimens (Spirit 26b)

Participants will be given the option to provide written consent for their blood samples and related meta-data to be included in an anonymous manner in a biobank.

### Intervention for RCT 1 (Spirit 6b and 11a)

The two arms in RCT 1 are: (a) the intervention arm, which is a package that includes food provision designed to supply the majority of estimated energy requirements for participants, combined with recipes, weekly meal plans, access to web-based nutrition education and opt-in online social support; and (b) the control arm, which is usual dietary intake and lifestyle, i.e., usual care.

The food provision will comprise food delivery to the participants’ homes for 12 weeks and is provided free of charge. The food will be assembled and delivered by two commercial companies, one of which is a meal kit home delivery service and the other is a grocery provider. The meal kit home delivery service will deliver food and recipes for five evening meals and fruit, and the grocery provider will deliver food that can be used for breakfast, lunch and snacks.

The food from both companies will be predominantly plant-based and rich in fruit and vegetables, grains, legumes, seafood, nuts, with some meat and dairy. The five New Zealand food groups used as the basis of the food provision are: vegetables; fruit; grain foods; legumes, nuts, seeds, fish and other seafood, eggs, poultry or red meat with fat removed; and reduced-fat milk and milk products. The amount of food provided is designed to provide approximately 75% of estimated energy requirements for the index participant and up to five members of their household/whānau. The food provided will align with a Mediterranean dietary pattern and when combined with the remaining ~25% energy requirements provided by participants themselves, will aim to fall within the acceptable total energy and macronutrient reference ranges for fat, carbohydrate and protein (32). Further details on the estimated energy, macronutrient distribution and food provided will be published separately. If a participant and their household/whānau go on holiday during the 12-week intervention period, the food delivery will be temporarily paused and will resume when they return home. During this time, they will be encouraged to adhere to the dietary pattern and will continue to have access to the education and social support resources.

The meal kit home delivery service and the grocery provider deliver food that has come from two sources; food provided in-kind to the study by New Zealand food and beverage (F&B) companies and food that is purchased by the research team. The food provided in-kind will be transported from the F&B companies directly to the meal kit home delivery service and the grocery provider, and then distributed to study participants. The involvement of the F&B companies was such that a nationwide call was made by the funding body (High Value Nutrition National Science Challenge) to F&B companies in New Zealand who wished to partner in the research study. The researchers assessed whether their foods align with the dietary pattern and if so, agreed on the quantity of food to be provided. The F&B companies had no direct role in the design or composition of the dietary pattern. Regular communication will take place between the researchers and the F&B companies who provide food.

The meal kit home delivery service will deliver meals that will have been pre-selected on behalf of the participants by a study dietitian. These meals will be either vegetarian, fish-based, or contain lean meat wherever possible. There are three grocery boxes that will be provided weekly by the grocery provider which have been developed by the research team to align with weekly breakfast, lunch and snack suggestions and recipes. These will be rotated over three weeks to provide variety, with each box being provided four times over the 12-weeks. An additional ‘Starter Box’ of products will be provided at the beginning of the 12-week period. The ‘Starter Box’ will include items that participants can use either immediately or throughout the entire 12-week period such as macadamia nuts, dried fruit, fish oil dressing, honey and seaweed snacks. Examples of the items in the other three boxes that are provided in a rotating manner are breakfast cereal, reduced-fat milk, seeds, rice cakes, olive oil, tuna, nuts, beans, lentils and fruit. The menu plans and recipes will be accessible through the study website.

The implementation of the dietary intervention incorporates elements of behavior change science which will be delivered by a multidisciplinary team including trained research staff and allied health care professionals including dietitians. Each site will have one research assistant and a doctoral student to support the clinic visits and arrange for the provision of food. The site staff will also make participants aware of the website and social media support options available to them and will play the website video titled “Introduction to the diet” so participants will be aware of what the dietary pattern entails. The behavioral aspects of the trial aim to support adherence to the NZMedDiet pattern and will include private Facebook groups and Facebook Messenger chats with a dietitian and peer support (online social support) which will be managed by one doctoral student who is a registered dietitian; a behavior change contract signed by participants prior to beginning RCT 1; and educational and motivational resources hosted on a participant facing website and YouTube channel. The website will be developed prior to the trial starting and will have six tabs covering: 1. Research Team, 2. Industry partners, 3. Nutrition support 4. Industry products, 5. Participant frequently asked questions and 6. Contact Us. The Nutrition Support section will link to short videos which will cover an introduction to the diet, goal setting, sample menus and recipes. The Nutrition support will also include multiple one-minute videos recorded by members of the research team, such as those entitled “Planning your shopping,” “Meal Planning,” “Getting to know your ingredients,” “Cooking” and “What to do with leftover food/ kai,” “Understanding your health data,” “Keeping motivated” and “Healthy Holiday Tips.” The behavior change wheel was used to design these resources to overcome identified barriers to the behavior, such as time and cost (17, 18). The participants will be able to interact with the website and online social support as little or as much as they chose.

### Control arm for RCT 1; usual dietary intake and lifestyle

Participants allocated to the control arm will continue their usual lifestyle and diet for 12-weeks. Nutritional education and social support will not be provided. At the end of this 12-week period, they will receive the intervention package described above, for 12-weeks.

### Intervention for RCT 2 (Spirit 6b and 11a)

At the end of the 12-weeks of RCT 1, participants randomized to the RCT 1 intervention will immediately be randomized to continuing the opt-in online social support or stopping it. Participants randomized initially to the RCT 1 control will then receive the RCT 1 intervention package described above for 12-weeks and will then be randomized to RCT 2 to either continuing the opt-in online social support or stopping it (Figure 1). All participants will continue to have access to the bespoke study website housing nutrition information.

### Criteria for discontinuing or modifying allocated interventions (Spirit 11b)

Enrolled participants from either arm can withdraw from the study at any time during the study. The reason for withdrawal will be captured.

### Strategies to improve adherence to the intervention (Spirit 11c)

Ongoing support will be provided by a member of the research team which includes the development and provision of additional short videos for the study website containing strategies around adhering to the intervention during holiday periods such as Christmas and New Year. Participants will also receive ongoing bespoke individualized support from the same team member in response to barriers they may encounter and queries they may have. Measurements and outcomes (Spirit 12).

### Outcomes for RCT1 and RCT2

The primary outcome for RCT 1 and RCT 2 is MetSSS in index participants. Secondary outcome measures for RCT 1 and RCT 2 for index and household/whānau participants are listed in Table 4. The behavior change elements of the trial are designed to support adherence to the dietary intervention and will be assessed using the Food Frequency Questionnaire (FFQ) and the Mediterranean adherence diet score which is taken from the FFQ data. While online engagement metrics as count scores will be assessed, these have little to no clinical relevance and will be used as modifying factors in the analysis.

**Table 4:** Primary and secondary outcome measures for RCT1, RCT2 and the longitudinal cohort study for index participants and for household/whānau members.

Outcome variables	RCT 1	RCT 2	LCS	Proposed analysis
Primary outcome				
MetSSS	X	X	X	ANCOVA with baseline measurements as covariate, see text for details of sensitivity analysis confounders and sub-group
Secondary outcomes				
Weight	X	X	X	ANCOVA with baseline measurements as covariate
Body mass index	X	X	X	ANCOVA with baseline measurements as covariate, consideration of logarithm transformation
Lean mass (FFM calculated from BIA and DXA measurements)	X	X	X	ANCOVA with baseline measurements as covariate
Fat mass (calculated from BIA and DXA Measurements)	X	X	X	ANCOVA with baseline measurements as covariate
Dietary pattern measured using Food Frequency Questionnaire	X	X	X	
Individual components of MetSSS				
Glucose	X	X	X	ANCOVA with baseline measurements as covariate
Triglycerides	X	X	X	ANCOVA with baseline measurements as covariate, consideration of logarithm transformation
HDL cholesterol	X	X	X	ANCOVA with baseline measurements as covariate
Systolic blood pressure	X	X	X	ANCOVA with baseline measurements as covariate
Waist circumference	X	X	X	ANCOVA with baseline measurements as covariate
HbA1c	X	X	X	ANCOVA with baseline measurements as covariate
Insulin	X	X	X	ANCOVA with baseline measurements as covariate, consideration of logarithm transformation
HOMA	X	X	X	ANCOVA with baseline measurements as covariate, consideration of logarithm transformation
Total cholesterol	X	X	X	ANCOVA with baseline measurements as covariate
LDL cholesterol	X	X	X	ANCOVA with baseline measurements as covariate
Diastolic BP	X	X	X	ANCOVA with baseline measurements as covariate
hsCRP	X	X	X	Mann–Whitney test with Hodges Lehmann estimator
Quality of life				
SF36 (MCS and PCS and all dimensions)	X	X	X	ANCOVA with baseline measurements as covariate
Kaupapa Māori Wellbeing questionnaire	X	X	X	Analyzed and reported separately
Online engagement metrics	X	X		*Count scores*
Self-evaluation COM Questionnaire	X	X	X	*Count scores*
Impact evaluation	X	X	X	*Qualitative thematic analysis*

LCS, longitudinal cohort study; MetSSS, metabolic syndrome severity score; FFM, fat free mass; BIA, bioelectrical impedance; DXA, dual-energy X-ray absorptiometry; HDL, high density lipoprotein; HOMA, homeostatic model assessment; LDL, low density lipoprotein; hsCRP, high sensitivity C reactive protein; MCS, mental component summary; PCS, physical component summary.

#### Outcomes for longitudinal cohort study

The primary outcome measure for the longitudinal cohort study is MetSSS in index participants 52 weeks after the start of the RCT 1 intervention package, whether that be at the beginning of the trial for those randomized to this arm initially, or after the initial 12-week control period for those randomized to the usual care arm of RCT 1. Secondary outcome measures for index and household/whānau participants are also listed in Table 4.

#### Details of outcome measures

Clinical assessment including anthropometry, blood sample provision and bioelectrical impedance (BIA) or dual x-ray absorptiometry (DXA) scans will be carried out at the research sites as described in Table 5. Height (cm) will be measured using a wall mounted stadiometer and weight (kg) will be measured using electronic scales. Body composition to include fat mass and lean mass in kg and as %body weight will be measured using either DXA and/or BIA, depending on what is available at the site. Waist circumference (cm) will be measured using a non-stretch tape and blood pressure (BP) (mmHg) will be measured using an automated blood pressure machine. The mean of three measurements of height, weight and waist circumference will be used for these outcome variables. For BP, three measurements will be recorded and the mean of the second and third measurements will be used. Adherence to the dietary pattern will be measured during the intervention period using a 24-h dietary recall and a FFQ (25). The FFQ includes additional questions specifically related to the dietary pattern of interest. The dietary pattern will be assessed using the FFQ data to create a Mediterranean diet adherence score based on the number of serves of olive oil, vegetables, fruit, breads & cereals, legumes, nuts, fish and seafood, eggs, poultry, dairy foods, red meat and sweets that the participants consume (33). A fecal sample will be collected from index participants for future microbiome analysis (Table 5).

**Table 5:** Schedule of measurements for index participants.

Study period timepoints								
	Eligibility	Enrolment	Intervention and follow up					
	Visit 1 Screening	Visit 2 Baseline Enrolment	Phone contact	Email Contact	Visit 3a Control only	Visit 3b	Visit 4	Visit 5
RCT1 control group		Week 0			Week 12	Week 24	Week 36	Week 64
RCT1 intervention group			Week 2	Week 6		Week 12	Week 24	Week 52
Eligibility screen	✓							
Informed consent	✓							
Medical history	✓							
Education about RCT 1 and RCT 2		✓						
Questionnaires								
Otago short-form FFQ		✓			✓	✓	✓	✓
24 h dietary recall		✓		✓	✓	✓	✓	✓
IPAQ Physical activity		✓			✓	✓	✓	✓
Kaupapa Māori Wellbeing questionnaire		✓			✓	✓	✓	✓
Three factor eating questionnaire		✓			✓	✓	✓	✓
Gastrointestinal symptom rating scale		✓			✓	✓	✓	✓
Self-Evaluation COM questionnaire		✓			✓	✓	✓	✓
Impact evaluation for nutrition and behavior change support					✓	✓	✓	✓
SF-36 quality of life		✓			✓	✓	✓	✓
Assessments								
Height	✓							
Clinical measurements^a^	✓	✓			✓	✓	✓	✓
Body composition		✓			✓	✓	✓	✓
Current medications	✓	✓		✓	✓	✓	✓	✓
Adverse events			✓	✓	✓	✓	✓	✓
Fasting blood samples ^b^	✓	✓			✓	✓	✓	✓
Fecal sample		✓			✓	✓	✓	

^a^Weight, waist circumference, height, blood pressure in triplicate. ^b^HbA1c, fasting glucose, lipids and metabolites and hormones related to metabolic health Household/whanau members will attend visits 2, 3a/3b, 4, and 5.

A number of sub-studies may be undertaken which include consumer insights including that of Pacific island participants; mixed meal tolerance tests; use of a phone application for the measurement of dietary intake; fecal microbiome analyses. Details of these sub-studies will not be described in this paper.

### Participant timeline (Spirit 13)

The duration of the study is 52-weeks after the intervention component of RCT 1.

### Sample size (Spirit 14)

The sample size uses a clinically important difference of 0.4 for the MetSSS score based on detecting a ‘moderate’ effect size. This is because the MetSSS score was designed to represent the total number of standard deviations different for an overall risk factor profile for poor cardiovascular outcomes (19, 34). The standard deviation for the MetSSS for the calculation, 0.83, was derived from a large Auckland-based cohort study of these risk factors in a similar sample to that for recruitment (20). A t-test based sample size calculation used 90% power and a two-sided type I error rate of 0.05 was for 184 total participants and increased to 200 index participants to allow for a 10% non-completion rate. A smaller effect size is likely to be detectable by using baseline MetSSS as a co-variate in an ANCOVA.

### Assignment of interventions

#### Allocation sequence generation in RCT 1 and in RCT 2 (Spirit 16a)

Index participants and their household/whānau will be block randomized into RCT 1 at site level to either start the intervention package or continue usual dietary intake for 12 weeks. If there is a second person within the household/whānau who meets the criteria for an index participant, they will be allocated to the same arm as the first index participant, where applicable. Index participants will be randomized using a computer-generated sequence. Household/whānau members will be randomized to the same arm as their corresponding index participant. Index and household/whānau participants will all be randomized once their data have been collected at Visit 2. It is not possible to blind index participants or their household/whānau members to the intervention in RCT 1. Nor is it practical to blind study staff collecting data, however the principal investigator, study statistician and the laboratory team will remain blinded throughout the study and analysis period. Participants will be informed of which arm of RCT 2 they are randomized to after they have completed the RCT 1 intervention.

#### Concealment mechanism (Spirit 16b)

Participants will be randomized after eligibility and written consent has been confirmed at visit 1 and baseline measurements have been completed (index participants) and visit 2 (household/whānau participants).

#### Implementation (Spirit 16c)

A member of the study team will log into the online system to seek randomization. The allocated arm will be recorded in the participant’s file and in the Site File.

### Assignment of interventions: blinding

#### Who will be blinded (Spirit 17a)

The Principal investigator, laboratory technicians and study statistician will be blinded.

#### Procedure for unblinding if needed (Spirit 17b)

Not applicable.

### Data collection and management

#### Plans for assessment and collection of outcomes (Spirit 18a)

In addition to clinical measurements and blood samples, data will also be collected using participant-completed questionnaires on paper and/or electronically through the study period (Table 5). Data will be collected during interviewer-led assessments at the relevant timepoints, and during the phone calls. Body composition using a DXA scan and/or BIA will be measured in index participants only.

#### Plan to promote participant retention and complete follow-up (Spirit 18b)

If randomized to the intervention, participants may opt out of intervention activities that are offered but remain enrolled in the study and continue with clinical assessments, complete questionnaires and allow researchers access to their medical records. Index and household/whānau participants will receive phone, text message or email reminders regularly to complete the questionnaires, depending on their preference.

#### Data management (Spirit 19)

When an index participant consents to take part, they will be allocated a unique participant identification number. A corresponding household/whānau participant will be allocated a unique participant identification number which connects to the identification number of their corresponding index participant. Consent forms and other paperwork containing personal identifiable data including completed paper questionnaires will be stored in a locked filing cabinet or password protected computer at each site. Data collected on REDCap will be stored within the REDCap system. Dietary intake data collected on Intake 24 is stored on that platform. Personal and research data entered directly onto a computer by participants or by a member of the research team will only be accessible to members of the research team. Participant details will be anonymized in all publications and other means of dissemination that result from the trial.

#### Confidentiality (Spirit 27)

The principles of confidentiality will be adhered to. Data will be collected and retained in accordance with the Privacy Act 2020 and the National Ethics Advisory Committee National Ethical Standards for Health and Disability Research and Quality Improvement. Personal data will be kept only for as long as it is required. All data analysis will take place on encrypted, password-protected computers. No data will be released to any unauthorized third party without the written approval of Principal Investigator. Data will be available for monitoring by the Health and Disability Ethics Committee or regulatory agencies if requested. An archiving plan will be developed for all study materials in accordance with the Sponsor’s archiving policy, and study materials will be archived for 10 years from the end of the trial. Access to the protocol, data and the statistical code will be available through application to the Principal Investigator once the primary manuscripts have been submitted.

#### Plans for collection, laboratory evaluation and storage of biological specimen for genetic or molecular analyses in this trial/future use

Analysis of blood samples will be limited to analytes that will give information on the cardiometabolic health of participants. This will include but are not limited to HbA1c, lipids, glucose, insulin and high sensitivity C reactive protein. Participants will be given the option to provide consent for their blood and fecal samples to be included in an anonymous manner in a biobank.

### Statistical analyses

The primary analysis will be conducted according to the intention to treat principle. Appropriate data descriptions and plots will be used for all variables. Change from baseline variables will also be described as paired differences but, in general, baseline values of response variables will be used as covariates in analyses rather than analyzing change from baseline. For RCT 1 and RCT 2 and for the index participants, the primary analysis for the primary outcome variable will be Analysis of Covariance (ANCOVA) with the baseline value of the MetSSS as a continuous covariate and the randomized treatment as the main explanatory variable. This strategy will also be used for all continuous outcome variables with the appropriate baseline value of any specific outcome variable as the covariate. The baseline measurement will be just before the relevant randomization. Normal distribution assumptions for residuals will be checked for models of continuous outcome variables and if these are badly violated transformations, such as a logarithm transformation, will be used. For some variables, outlined in Table 4, it is already established that the logarithm transformation is likely to be appropriate. Where appropriate transformations cannot be identified a rank-based procedure, the Mann–Whitney test with the Hodges-Lehmann estimator of location difference, will be used. For the primary outcome variable, the MetSSS, a sensitivity analysis will be a secondary analysis adjusting for possible confounding by important explanatory variables that may have not been evenly distributed by the randomization. These will be as a composite categorical variable for whether medication was changed for any of blood pressure control, glucose management, or lipid control; and separately, for physical activity assessed by the international physical activity questionnaire score. Analysis will be by ANCOVA with these confounding variables in addition to the baseline MetSSS and randomized treatment. Another secondary analysis will be to explore if randomized treatment allocation is associated with different effects in subgroups; using an appropriate treatment by subgroup interaction models. These subgroups are: age, sex, baseline diet adherence score, household size, and whether a household has children. For the continuous variables the analysis of the interaction will be on the continuous scale but for illustrative purposes, and for use in a Forest-like plot, will also be estimated for the first and third quartile of the continuous predictor distribution. Another important secondary analysis will be of all participants treating each household as a cluster and using a Mixed Linear model with baseline measurement and randomized treatment as fixed effects, and household clusters as random effects. If in the event there are very few clusters, the random effect may not be estimable and then this will be equivalent to ANCOVA. The Intraclass correlation coefficient for the clustering effect will be estimated directly from the variance components in the mixed linear models, should a cluster effect be estimable. Categorical and ordinal variables will be analyzed by logistic regression, the latter corresponding to ordinal regression, for the individual participant analysis, and by a Generalized Mixed Linear Model for all participants. There may be other effect modifiers that we will consider but these will be outlined in the Statistical Analysis Plan.

For the longitudinal cohort study stage, a similar ANCOVA strategy will be used for the final measurement of the continuous outcomes. A mixed linear model will also be used to examine linear trends in changes in outcomes by accounting for correlation between measurements in the same participants as well as the clustering effect for an analysis of all participants.

SAS version 9.4 will be used for analyses.

### Oversight and monitoring

#### Composition of the coordinating center and the trial steering committee (Spirit 5d)

The study is led by a Senior Leadership Team who meet every two weeks and will be responsible for the oversight of the trial. It is composed of the Principal Investigator and senior members of the research team. The Operations Team meets every two to four weeks and will be responsible for the day-to-day running of the trial. In addition, author FEL operates as research program manager and works closely with all sites and with food providers. The trial also has an independent Māori advisory committee providing oversight to support the research team, Māori researchers and Māori participants.

### Safety

As per standard procedure, any changes to the protocol or serious adverse events will be communicated to the Health and Disability Ethics Committee. Any significant changes in the protocol will be notified to participants by study staff, and any changes to the informed consent will be actioned at the next scheduled study visit. There is no Data Monitoring Committee for this trial and The Sponsor is responsible for monitoring the conduct of the trial.

## Discussion

This trial has been designed to investigate the effect of the Mediterranean dietary pattern incorporating high quality New Zealand foods, implemented using elements of behavior science, on the cardiometabolic health of people in Aotearoa New Zealand. The trial has a number of strengths, the first of which relates to the trial design which is an efficient way to answer three separate but linked important questions which is important in the reduction of participant burden and overall research costs. It comprises two RCTs and a longitudinal cohort study which allows the effect of the 12-week combined intervention package (RCT 1) and then subsequent ongoing online social support (RCT 2) to be tested. This is then followed by testing of whether any dietary changes and effects on cardiometabolic health are maintained at 12 months through a longitudinal cohort study.

The inclusion of household/whānau participants, including any children over 5 years in the household/whānau is important when considering the impact of the dietary intervention on cardiometabolic health, where shared genetics and/or environmental factors influence health outcomes. Not only do household/whānau participants receive the study food, which is likely to improve uptake and adherence for the index participant, but the household/whānau participants are also included in the measurements, allowing an assessment of whether their health status can also be improved. Furthermore, in this study we are providing a substantive proportion of the whole household/whānau weekly food requirements in order to reduce barriers to change from cost and availability, and to increase the opportunity for trying new foods and facilitate change in dietary pattern. While other studies have provided food to facilitate adherence to an intervention (13, 35), the current study provides up to 75% of estimated energy requirements across all food groups in a way that aligns with the NZMedDiet pattern. We have demonstrated that the approach of both a household/whānau based intervention and the provision of food for a whole diet intervention is acceptable and culturally relevant for people in Aotearoa New Zealand in our feasibility study (23).

The current trial has also taken a broad collaborative approach in a significant partnership between academia and the food industry and aims to provide locally produced food. The study team has established relationships with over thirty food companies and negotiated the provision of food that aligns with the dietary pattern. Importantly, although contributing foods and beverages that align with the NZMedDiet pattern, the food industry has not been involved with or influenced the trial design and will not be involved with the data analysis or reporting of the trial findings. The goal of the funding body, High Value Nutrition National Science Challenge, is to bring together academics and the food industry to grow science excellence and knowledge to deliver food to the world which is associated with positive health outcomes. The relationships built are important and demonstrate an ability for academics and industry to work together for better health for communities. Food grown and produced in Aotearoa New Zealand is known to be nutritious and of high quality, and foods which align with the MedDiet dietary pattern can be predominantly sourced in Aotearoa New Zealand. In this trial, engagement and partnership with the local food industry to provide food for the participants allows the provision of locally sourced, seasonable and fresh food.

Another key component of this trial is the importance of Māori as the indigenous New Zealanders across the trial. This includes Māori as co-investigators on the Science Leadership Team, one study site being based at Kokiri Marae where Māori participants will be recruited using local protocols for engagement. The trial includes Māori research staff, Māori F&B companies and Māori participants in the study. The trial also has an independent Māori committee providing oversight to support the research team, Māori researchers and Māori participants.

The use of the MetSSS as the primary outcome measure was carefully considered as the dietary intervention may have small but collectively important effects on multiple risk factors for cardiometabolic disease. Therefore, a composite outcome measure which incorporates several of these, rather than choosing one specific risk factor, is attractive. MetSSS is a continuous variable derived from the traditional components of the metabolic syndrome (20, 36). The MetSSS has been shown to predict future cardiometabolic disease and can be modified by lifestyle including diet and exercise, and pharmacological interventions to corresponding change in cardio-metabolic disease risk. It is therefore a useful tool in assessing the impact of a dietary intervention on metabolic health (19). In an Aotearoa New Zealand population, the MetSSS positively associated with impaired glucose regulatory status and history of cardiovascular disease for all ethnic groups (20, 21). This places it in a good position to have utility as a tool to quantify an individual’s cardiometabolic disease risk within the multi-ethnic population of Aotearoa New Zealand (20).

There are several challenges faced by the study team to effectively deliver this trial. The feasibility study has shown that it is possible to recruit individuals and household/whānau to a whānau-based intervention. It also showed that the two-step screening process was effective and efficient and that the commercial meal kit home delivery service was acceptable. However, in the current trial we also need to bring together F&B providers, co-ordinate collection and delivery of these foods integrated into the dietary pattern and further enable and facilitate adoption and adherence to this. Informed by the feasibility study we have incorporated more behavior change support and further information on how to prepare or use foods which participants may not have encountered before. One particular challenge will be to maintain participants in the trial after the provision of food has finished and adoption of the dietary pattern when they are purchasing all of their own food. This is one of the important research questions being addressed; whether substantive food provision and support enables subsequent sustained dietary pattern change.

In summary, this trial combines two RCTs and a longitudinal cohort study to investigate whether a household/whānau-based dietary intervention incorporating high quality whole foods from New Zealand, consistent with a Mediterranean dietary pattern, combined with dietary change support, improves the cardiometabolic health of New Zealanders.

## Data availability statement

The original contributions presented in the study are included in the article/supplementary material, further inquiries can be directed to the corresponding author.

## Ethics statement

Ethical approval was granted by the New Zealand Health and Disability Ethics Committee – Northern B branch – reference 2022 FULL 12045. Dissemination is planned in a series of journal articles, conference abstracts and presentations; reports and presentations to industry partners; communication with study participants and the wider community.

## Author contributions

FL: Writing – original draft, Writing – review & editing. AP: Writing – original draft, Writing – review & editing. AB: Writing – original draft, Writing – review & editing. AW: Writing – review & editing, Writing – original draft. MF: Writing – review & editing. AR: Writing – review & editing. CD: Writing – review & editing. JM: Writing – review & editing. CR: Writing – review & editing. DC: Writing – review & editing. TM: Writing – review & editing. RG: Writing – review & editing. MW: Writing – review & editing. JK: Writing – original draft, Writing – review & editing.

## Footnotes

^1^ www.hrwp.co.nz

## References

[1] AKR. Custom requested mortality dataset provided to Heart Foundation from the NZ mortality collection. Wellington: Ministry of Heath (2021).

[2] GBD 2017 Diet Collaborators. Health effects of dietary risks in 195 countries, 1990-2017: a systematic analysis for the global burden of disease study 2017. Lancet. (2019) 393:1958–72. doi: 10.1016/S0140-6736(19)30041-8 30954305 PMC6899507

[3] Martínez-González MA García-Arellano A Toledo E Salas-Salvadó J Buil-Cosiales P Corella D . A 14-item Mediterranean diet assessment tool and obesity indexes among high-risk subjects: the PREDIMED trial. PLoS One. (2012) 7:e43134. doi: 10.1371/journal.pone.0043134, PMID: 22905215 PMC3419206

[4] Diotallevi C Fava F Gobbetti M Tuohy K. Healthy dietary patterns to reduce obesity-related metabolic disease: polyphenol-microbiome interactions unifying health effects across geography. Curr Opin Clin Nutr Metab Care. (2020) 23:437–44. doi: 10.1097/MCO.0000000000000697, PMID: 32941185

[5] Munt AE Partridge SR Allman-Farinelli M. The barriers and enablers of healthy eating among young adults: a missing piece of the obesity puzzle: a scoping review. Obesity Rev. (2017) 18:1–17. doi: 10.1111/obr.12472, PMID: 27764897

[6] Lovell AL Roy R Klein A Cavadino A Foster M Krebs JD . Habitual dietary patterns, nutrient intakes, and adherence to the Mediterranean diet among New Zealand adults: the NZ MED cross-sectional study. Nutrients. (2023) 15:2663. doi: 10.3390/nu15122663, PMID: 37375568 PMC10303228

[7] Beck KL Jones B Ullah I McNaughton SA Haslett SJ Stonehouse W. Associations between dietary patterns, socio-demographic factors and anthropometric measurements in adult new Zealanders: an analysis of data from the 2008/09 New Zealand adult nutrition survey. Eur J Nutr. (2018) 57:1421–33. doi: 10.1007/s00394-017-1421-3, PMID: 28378296

[8] Murphy KJ Parletta N. Implementing a Mediterranean-style diet outside the Mediterranean region. Curr Atheroscler Rep. (2018) 20:28. doi: 10.1007/s11883-018-0732-z, PMID: 29728772

[9] Timlin D McCormack JM Kerr M Keaver L Simpson EEA. Are dietary interventions with a behaviour change theoretical framework effective in changing dietary patterns? A systematic review. BMC Public Health. (2020) 20:1857. doi: 10.1186/s12889-020-09985-8, PMID: 33272230 PMC7713327

[10] Gough B Conner MT. Barriers to healthy eating amongst men: a qualitative analysis. Soc Sci Med. (2006) 62:387–95. doi: 10.1016/j.socscimed.2005.05.032, PMID: 16011867

[11] Coppell KJ Abel SL Freer T Gray A Sharp K Norton JK . The effectiveness of a primary care nursing-led dietary intervention for prediabetes: a mixed methods pilot study. BMC Fam Pract. (2017) 18:106. doi: 10.1186/s12875-017-0671-8, PMID: 29268719 PMC5740796

[12] Abel SL Whitehead LC Tipene-Leach DC Coppell KJ. Proximal and distal influences on dietary change among a diverse group with prediabetes participating in a pragmatic, primary care nurse-led intervention: a qualitative study. Public Health Nutr. (2021) 24:6015–26. doi: 10.1017/S1368980021001968, PMID: 33966689 PMC11148607

[13] Jeffery RW Wing RR Thorson C Burton LR Raether C Harvey J . Strengthening behavioral interventions for weight loss: a randomized trial of food provision and monetary incentives. J Consult Clin Psychol. (1993) 61:1038–45. doi: 10.1037/0022-006X.61.6.1038, PMID: 8113481

[14] Francis H Carryer J Wilkinson J. The complexity of food for people with multiple long-term health conditions. J Prim Health Care. (2018) 10:186–93. doi: 10.1071/HC18020, PMID: 31039931

[15] Berge JM Everts JC. Family-based interventions targeting childhood obesity: a meta-analysis. Child Obes. (2011) 7:110–21. doi: 10.1089/chi.2011.07.02.1004.berge, PMID: 26182126 PMC4504253

[16] Fraser K Love P Campbell KJ Ball K Opie RS. Meal kits in the family setting: impacts on family dynamics, nutrition, social and mental health. Appetite. (2022) 169:105816. doi: 10.1016/j.appet.2021.105816, PMID: 34801628

[17] Michie S van Stralen MM West R. The behaviour change wheel: a new method for characterising and designing behaviour change interventions. Implement Sci. (2011) 6:42. doi: 10.1186/1748-5908-6-42, PMID: 21513547 PMC3096582

[18] Michie S. Implementation science: understanding behaviour change and maintenance. BMC Health Serv Res. (2014) 14:O9. doi: 10.1186/1472-6963-14-S2-O9

[19] DeBoer MD Filipp SL Gurka MJ. Use of a metabolic syndrome severity Z score to track risk during treatment of prediabetes: an analysis of the diabetes prevention program. Diabetes Care. (2018) 41:2421–30. doi: 10.2337/dc18-1079, PMID: 30275282 PMC6196828

[20] Merry TL Metcalf P Scragg R Gearry R Foster M Krebs JD. Metabolic syndrome severity score (MetSSS) associates with metabolic health status in multi-ethnic Aotearoa New Zealand cohorts. Diabetes Res Clin Pract. (2022) 192:110088. doi: 10.1016/j.diabres.2022.110088, PMID: 36154929

[21] Barthow C Pullon S Weatherall M Krebs J. They’re sicker than we think: an exploratory study profiling the cardio-metabolic health in a sample of adults with pre-diabetes in Aotearoa New Zealand. J Prim Health Care. (2022) 14:221–8. doi: 10.1071/HC22068, PMID: 36178844

[22] Chan AW Tetzlaff JM Altman DG Laupacis A Gøtzsche PC Krleža-Jerić K . SPIRIT 2013 statement: defining standard protocol items for clinical trials. Ann Intern Med. (2013) 158:200–7. doi: 10.7326/0003-4819-158-3-201302050-00583, PMID: 23295957 PMC5114123

[23] Parry-Strong A Gearry R Merry TL Weatherall M Davies C Worthington A . High Quality Aotearoa New Zealand Dietary Pattern adapting a Mediterranean Diet for Metabolic Health: a feasibility study. BMC Nutrition [In press]. 10.1186/s40795-023-00805-x PMC10709956 38066654

[24] Martin A Neale EP Tapsell LC. The clinical utility of the AUSDRISK tool in assessing change in type 2 diabetes risk in overweight/obese volunteers undertaking a healthy lifestyle intervention. Prev Med Rep. (2019) 13:80–4. doi: 10.1016/j.pmedr.2018.11.020 30534513 PMC6282634

[25] Sam CHY Skidmore P Skeaff S Wall C Bradbury KE Parackal S. Relative validity and reproducibility of a short food frequency questionnaire to assess nutrient intakes of New Zealand adults. Nutrients. (2020) 12:619. doi: 10.3390/nu12030619, PMID: 32120797 PMC7146506

[26] Stunkard AJ Messick S. The three-factor eating questionnaire to measure dietary restraint, disinhibition and hunger. J Psychosom Res. (1985) 29:71–83. doi: 10.1016/0022-3999(85)90010-8 3981480

[27] Craig CL Marshall AL Sjöström M Bauman AE Booth ML Ainsworth BE . International physical activity questionnaire: 12-country reliability and validity. Med Sci Sports Exerc. (2003) 35:1381–95. doi: 10.1249/01.MSS.0000078924.61453.FB, PMID: 12900694

[28] Jenkinson C Stewart-Brown S Petersen S Paice C. Assessment of the SF-36 version 2 in the United Kingdom. J Epidemiol Community Health. (1999) 53:46–50. doi: 10.1136/jech.53.1.46, PMID: 10326053 PMC1756775

[29] Svedlund J Sjödin I Dotevall G. GSRS--a clinical rating scale for gastrointestinal symptoms in patients with irritable bowel syndrome and peptic ulcer disease. Dig Dis Sci. (1988) 33:129–34. doi: 10.1007/BF01535722, PMID: 3123181

[30] Keyworth C Epton T Goldthorpe J Calam R Armitage CJ. Acceptability, reliability, and validity of a brief measure of capabilities, opportunities, and motivations ("COM-B"). Br J Health Psychol. (2020) 25:474–501. doi: 10.1111/bjhp.12417, PMID: 32314500

[31] Michie S Abraham C Eccles MP Francis JJ Hardeman W Johnston M. Strengthening evaluation and implementation by specifying components of behaviour change interventions: a study protocol. Implement Sci. (2011) 6:10. doi: 10.1186/1748-5908-6-10, PMID: 21299860 PMC3041694

[32] Eat for Health. NZ Ministry of Health with the Australian Government National Health and Medical Research Council. (2018). Available at: https://www.eatforhealth.gov.au/nutrient-reference-values.

[33] Tong TYN Wareham NJ Khaw K-T Imamura F Forouhi NG. Prospective association of the Mediterranean diet with cardiovascular disease incidence and mortality and its population impact in a non-Mediterranean population: the EPIC-Norfolk study. BMC Med. (2016) 14:135. doi: 10.1186/s12916-016-0677-4, PMID: 27679997 PMC5041408

[34] Gurka MJ Lilly CL Oliver MN DeBoer MD. An examination of sex and racial/ethnic differences in the metabolic syndrome among adults: a confirmatory factor analysis and a resulting continuous severity score. Metab Clin Exp. (2014) 63:218–25. doi: 10.1016/j.metabol.2013.10.006 24290837 PMC4071942

[35] Dutton GR Laitner MH Perri MG. Lifestyle interventions for cardiovascular disease risk reduction: a systematic review of the effects of diet composition, food provision, and treatment modality on weight loss. Curr Atheroscler Rep. (2014) 16:442. doi: 10.1007/s11883-014-0442-0 25092578 PMC4157951

[36] Huang PL. A comprehensive definition for metabolic syndrome. Dis Model Mech. (2009) 2:231–7. doi: 10.1242/dmm.001180, PMID: 19407331 PMC2675814

